# Central nervous system involvement and mimickers in ANCA associated vasculitis

**DOI:** 10.1007/s00415-025-13203-z

**Published:** 2025-07-08

**Authors:** Yeliz Yagiz Ozogul, Sinem Nihal Esatoglu, Murat Ozogul, Osman Kizilkilic, Yesim Ozguler, Emire Seyahi, Serdal Ugurlu, Melike Melikoglu, Izzet Fresko, Vedat Hamuryudan, Ugur Uygunoglu, Gulen Hatemi

**Affiliations:** 1 Department of Internal Medicine, Cerrahpasa Medical School, Istanbul University-Cerrahpasa, Istanbul, Türkiye; 2 Division of Rheumatology, Department of Internal Medicine, Cerrahpasa Medical School, Istanbul University-Cerrahpasa, Istanbul, Türkiye; 3 Department of Radiology, University of Health Sciences, Haydarpasa Numune Training and Research Hospital, Istanbul, Türkiye; 4 Department of Radiology, Cerrahpasa Medical School, Istanbul University-Cerrahpasa, Istanbul, Türkiye; 5 Department of Neurology, Cerrahpasa Medical School, Istanbul University-Cerrahpasa, 34098 Istanbul, Türkiye

**Keywords:** Antineutrophil cytoplasmic antibodies, Central nervous system, ANCA associated vasculitis, Granulomatosis with polyangiitis, Microscopic polyangiitis, Eosinophilic granulomatosis with polyangiitis

## Abstract

**Objective:**

Central nervous system (CNS) involvement is rare in ANCA-associated vasculitis (AAV). On the other hand, AAV patients may develop complications or other conditions that mimic CNS involvement. We aimed to present the clinical, laboratory and imaging features of our AAV patients with CNS involvement and conditions other than CNS involvement that caused neurologic symptoms**.**

**Method:**

We surveyed the charts of 430 AAV patients in order to identify patients who were evaluated for neurologic symptoms suggesting CNS involvement. We extracted data on their demographics, AAV features, neurologic symptoms, final diagnoses after neurologic work-up, and their outcomes.

**Results:**

Of 430 AAV patients, 61 patients (14%) (41 GPA, 11 MPA, 9 EGPA; 27 women, 34 men; mean age: 51.6 ± 15.4 years) with neurologic symptoms were identified. At the time of the occurrence of neurologic symptoms, all patients had active disease [median (IQR) BVAS=11.9 (7–15)]. The causes of neurologic symptoms were CNS involvement of AAV in 7 patients (meningeal involvement in 3, ischemic cerebrovascular accident in 2, intracranial hypertension in 1, and cerebral venous sinus thrombosis in 1), other AAV manifestations such as ocular, orbital and nasopharyngeal involvement in 30, and drug-related adverse events or comorbidities mimicking CNS involvement such as infections, atherosclerotic or thromboembolic events in 15 patients. Neurologic work-up did not lead to an underlying condition in 9.

**Conclusion:**

CNS involvement was uncommon, observed in only 1.6% of patients. AAV manifestations other than CNS involvement, as well as complications like infections and cardiovascular disease may mimic CNS involvement in patients with AAV.

## Introduction

Antineutrophil cytoplasmic antibody (ANCA)–associated vasculitis (AAV) is characterized by necrotizing vasculitis primarily affecting small to medium-sized vessels and comprises granulomatosis with polyangiitis (GPA), microscopic polyangiitis (MPA), and eosinophilic granulomatosis with polyangiitis (EGPA) [1]. While AAV may affect various organs, the upper respiratory tract, lungs, and kidneys are the most commonly involved [2]. Age, pulmonary and renal involvement, and a high Birmingham vasculitis activity score (BVAS) are associated with poor prognosis among AAV patients [3]. One-year mortality rate due to AAV had decreased from 80 to 10% with the introduction of cyclophosphamide (CYC) and glucocorticoids (GC) for AAV treatment. Over the last decade, rituximab (RTX) has been shown to have comparable efficacy to CYC for both remission induction and maintenance [4–6].

The peripheral nervous system (PNS) is commonly affected in patients with AAV, with a frequency of 20–65%. On the other hand, central nervous system (CNS) involvement is relatively rare, reported in about 5–15% of cases. Although its association with increased mortality was not demonstrated, CNS involvement in AAV leads to significant morbidity in affected patients [7–9]. CNS involvement may occur due to ischemia secondary to vascular inflammation or granulomas, and neurologic symptoms may arise from the involvement of the brain parenchyma, meninges, pituitary gland, spinal cord, and/or cranial nerves [10].

There are only few previous studies that provide a comprehensive analysis of CNS involvement among AAV patients [9–14]. Moreover, various factors such as involvement of other organ systems, drug-related adverse events, infections, and comorbidities can mimic CNS involvement in patients with AAV. The extent to which these mimickers contribute to symptoms suggesting CNS involvement was not well studied. In this study, we aimed to describe the clinical, laboratory, and imaging features, and outcome of our AAV patients with CNS involvement and to identify conditions mimicking CNS involvement in AAV.

## Materials and methods

### Patients

We reviewed the medical records of 430 AAV patients admitted to our center between 2001 and 2022, to identify patients who were evaluated for neurologic signs or symptoms suggesting CNS involvement. We extracted data on demographic features, age at AAV diagnosis, age at onset of CNS manifestations, follow-up duration from AAV diagnosis to the last visit, initial symptoms attributable to AAV, AAV features including ANCA serology, types of organ involvement at disease onset and during the disease course, medications used, neurologic signs and symptoms, final diagnoses related to CNS following neurologic work-up, and outcome. The disease activity at the onset of CNS manifestations was assessed using BVAS [11].

### Definitions

AAV patients presenting with neurologic symptoms attributed to the involvement of the brain parenchyma, meninges, pituitary gland, spinal cord and/or cranial nerves by AAV were classified as having CNS involvement. The definition of CNS involvement was the presence of neurological symptoms or signs concomitant with vasculitis, not explained by other pathologies. Other causes were categorized as neurologic symptoms due to AAV manifestations other than CNS involvement, drug related adverse events or comorbidities such as infections, drug-related adverse events, or cardiovascular disease. If peripheral cranial nerve involvement was observed as a result of upper respiratory tract involvement or eye involvement, these cases were grouped as having neurologic symptoms due to AAV manifestations other than CNS involvement. An experienced neurologist (UU) reviewed the neurologic work-up findings including clinical, laboratory, and radiologic assessments, and made the final decision regarding the classification of CNS involvement. In active AAV patients who had neurologic symptoms but no objective neurologic signs on physical examination or imaging, and whose symptoms resolved following immunosuppressive therapy, we attributed these symptoms to overall disease activity.

Immunosuppressive agents that were used for AAV management in addition to glucocorticoids included RTX, CYC, azathioprine (AZA), mycophenolate mofetil (MMF), and intravenous immunoglobulin (IVIG).

### Statistical analysis

The statistical analyses were conducted using SPSS 20.0. Descriptive statistics were used to summarize the variables, with continuous variables presented as either mean ± standard deviation or median (interquartile range, IQR) depending on their relevance to the analysis.

## Results

Among the 430 AAV patients, 61 patients (14%) (41 GPA, 11 MPA, 9 EGPA; 34 men (56%); mean (SD) age: 51.6 (15.4) years) had neurologic signs or symptoms at some time during the disease course. Neurologic signs or symptoms were present at disease onset in 25 (41%) patients. The remaining 36 (59%) patients developed neurologic signs or symptoms within a mean (SD) follow up of 33.4 (40.4) months after AAV diagnosis. The most common neurologic signs or symptoms attributable to CNS involvement were headache (n = 20), muscle weakness (n = 17), numbness (n = 17), and visual impairment (n = 16). At the time of the occurrence of neurologic symptoms, all patients had active disease, with a median BVAS score of 11 (IQR: 7–15)]. Additionally, 21 patients (34%) had accompanying PNS involvement.

**Fig. 1:**
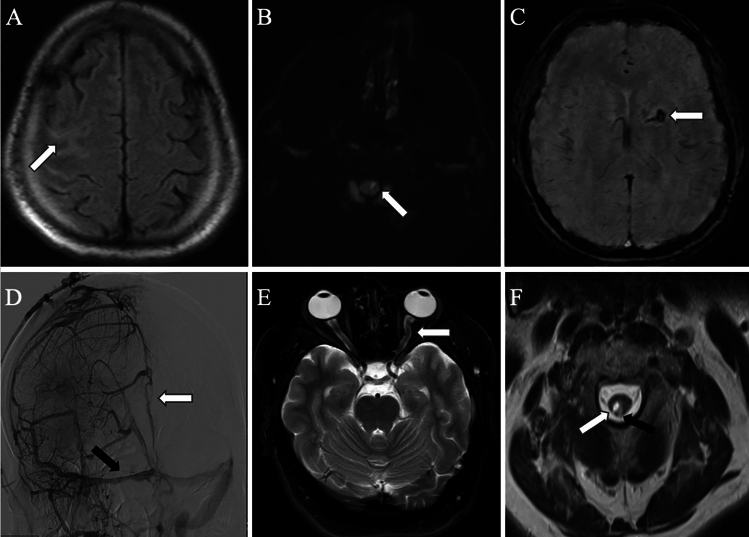
Causes for neurologic sign or symptoms suggesting CNS involvement among 61 AAV patients. **a** Sinonasal involvement in 4 patients, nasal involvement in 2 patients, nasopharyngeal mass and PNS involvement in 1 patient. **b** Orbital involvement in 1 patient, ocular involvement in 1 patient, retinal vasculitis in 1 patient, orbital and paranasal involvement, secondary facial paralysis and PNS in 2 patients. **c** Facial paralysis due to facial nerve involvement, orbital involvement and parotid gland involvement in 1 patient each. **d** Cardioembolic CVA due to cardiac mass and cardiac thrombus in 1 patient each. **e** Vertigo and nausea associated with CYC in 1 patient, subretinal fluid associated with glucocorticoid in 1 patient

A final diagnosis of CNS involvement of AAV was established in 7 (11.4%) patients. The neurologic symptoms were associated with AAV manifestations other than CNS involvement in 30 (49.1%) patients. The causes of neurologic symptoms were drug-related adverse events or comorbidities affecting the CNS in 15 patients (24.5%). The neurologic work-up did not reveal an underlying condition in 9 patients (15%). Causes for neurologic signs or symptoms and demographic data are presented in Fig. 1 and Table 1.

**Table 1:** Characteristics of the 61 AAV patients and causes for neurologic signs or symptoms

	AAV patients with neurologic signs or symptoms (n=61)
Male, n (%)	34 (56)
Mean ± SD age at the time of this study (years)	51.6 ± 15.4
Type of AAV, n (%)	
GPA	41 (67.2)
MPA	11 (18)
EGPA	9 (14.7)
CNS involvement of AAV, n (%)	7 (11.4)
Meningeal involvement	3 (4.9)
Meningeal involvement and ischemic CVA	1
Meningeal involvement, ischemic CVA and syringomyelia	1
Meningeal involvement	1
Vascular involvement	2
Ischemic CVA	1
Ischemic CVA with hemorrhagic transformation	1
Intracranial hypertension	1
Cerebral venous sinus thrombosis	1
AAV manifestations other than CNS involvement, n (%)	30 (49.1)
PNS involvement	8
Sinonasal involvement	4
Nasal involvement	2
Associated with overall disease activity*	2
Orbital and paranasal involvement, secondary facial paralysis and PNS	2
Orbital involvement	1
Ocular involvement	1
Facial paralysis due to facial nerve involvement	1
Facial paralysis due to orbital involvement	1
Facial paralysis due to parotid gland involvement	1
Associated with overall disease activity and PNS involvement	1
Associated with overall disease activity and cervical hernia	1
Retinal vasculitis	1
Nasopharyngeal mass and PNS involvement	1
Parotid gland involvement	1
Cardioembolic CVA associated with cardiac mass	1
Cardiac thrombus	1
Drug related adverse events or comorbidities, n (%)	15 (24.5)
Atherosclerotic CVA	5
Drug side effect	2
Cardioembolic CVA associated with atrial fibrillation and PNS inv.	1
PRES	1
Hemorrhagic CVA secondary to hypertension	1
Spondylodiscitis complicated with aortic pseudoaneurysm	1
Heart failure	1
Skull base osteomyelitis	1
Septic cranial embolism	1
Ketoacidosis and sepsis	1
Neurologic work-up did not lead to an underlying condition	9 (14.7%)

*AAV* ANCA associated vasculitis, *CNS* central nervous system, *CVA* cerebrovascular accident, *CVST* cerebral venous sinus thrombosis, *EGPA* eosinophilic granulomatosis with polyangiitis, *GPA* granulomatosis with polyangiitis, *MPA* microscopic polyangiitis, *PNS* peripheral nervous system

*In active AAV patients who had neurologic symptoms but no objective neurologic signs on physical examination or imaging, and whose symptoms resolved following immunosuppressive therapy, we attributed these symptoms to overall disease activity

### AAV patients with CNS involvement

Final diagnosis was CNS involvement of AAV in 7 (11.4%) patients (5 GPA, 1 MPA, 1 EGPA; 5 men). The frequency of CNS involvement for each type of AAV was 5/41 (12.1%) for GPA, 1/11 (9%) for MPA and 1/9 (11.1%) for EGPA. Mean (SD) age at the time of CNS involvement was 39.5 (12.1) years. The mean time from AAV diagnosis to the development of CNS involvement was 10 (range 0–23) months. Three patients presented with headache, muscle weakness, seizures, and numbness, two patients presented with blurred vision, one patient with hearing loss, and one with dizziness. Three patients also had PNS involvement. In two patients, CNS involvement was one of the initial manifestations of AAV. In the remaining five patients, CNS involvement occurred while they were on immunosuppressive therapy. One patient was on AZA, one patient on both RTX and CYC, one patient on both CYC and low-dose GC, one patient on CYC, and one patient on both AZA and low-dose GC.

Neurologic events due to CNS involvement were meningeal involvement in three patients. One of these patients had ischemic cerebrovascular accident (CVA) and another had both ischemic CVA and syringomyelia in addition to meningeal involvement. The neurologic events were vascular in two other patients, presenting with ischemic CVA and ischemic CVA together with hemorrhagic transformation. The remaining two patients had intracranial hypertension and cerebral venous sinus thrombosis in one patient each.

**Fig. 2:**
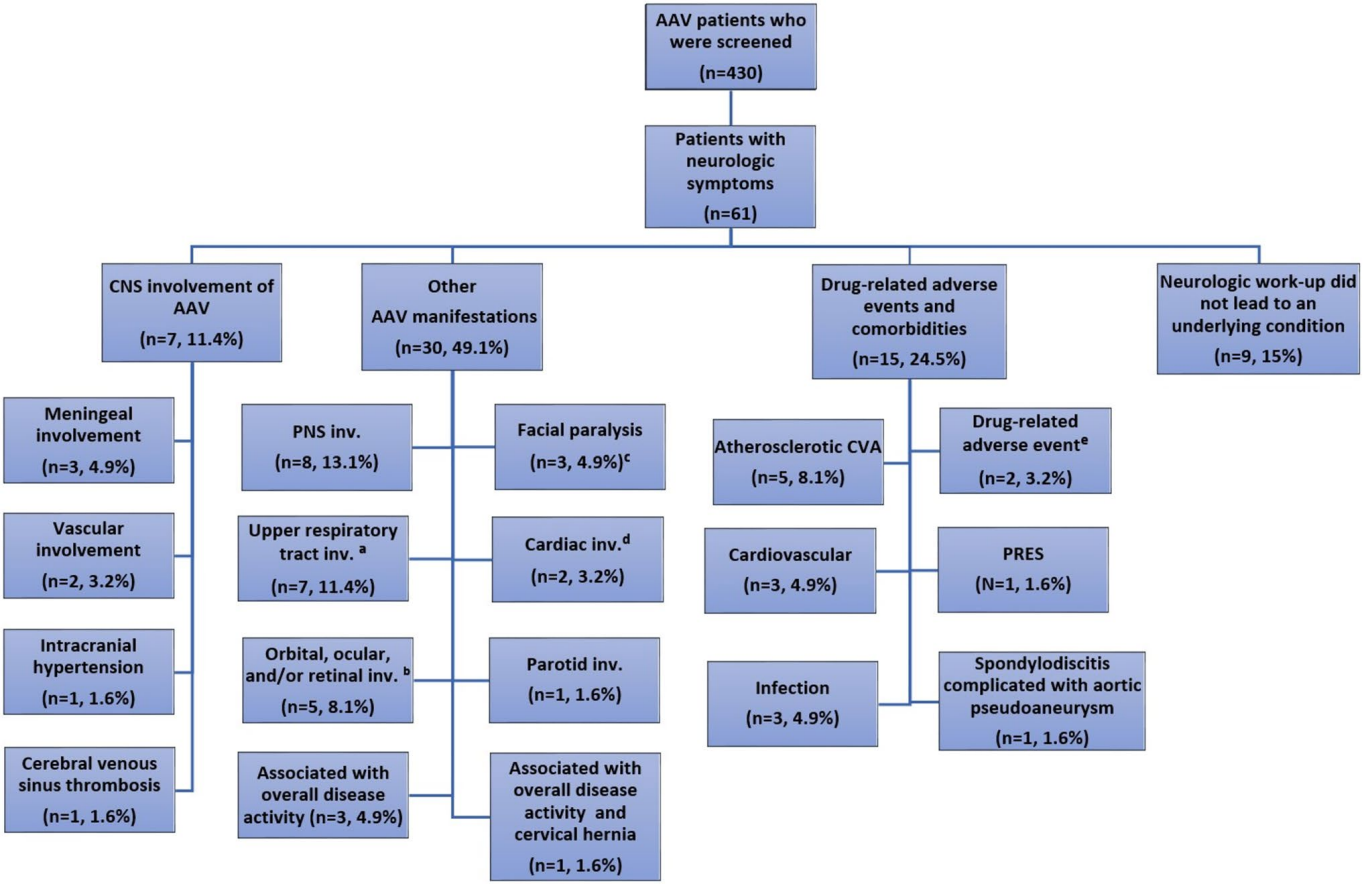
Radiological images of AAV patients with CNS involvement. **A** Axial Flair Image demonstrates right hemisphere meningeal involvement (arrow). **B** DWI demonstrates hyperintense area due to ischemia in brainstem (arrow). **C** SWI demonstrates hemorrhagic transformation in left internal capsule (arrow). **D** Right internal carotid artery selective DSA examination venous phase image shows occlusion and partial recanalization of the superior sagittal sinus (white arrow) as well as right transvers sinus (black arrow). **E** Axial T2W image demonstrates optic nerve sheath tortuosity due to intracranial hypertension (arrow). **F** Axial T2 weighted image shows syringomyelia in the right half of the spinal cord (white arrow) and associated increased signal in the dorsal aspect and left half of the spinal cord (black arrow) related with former myelitis. *AAV* ANCA associated vasculitis, *CNS* Central nervous system, *DSA* Digital subtraction angiography, *DWI* Diffusion weighted imaging, *MRV* Magnetic Resonance Venography, *SWI* Susceptibility weighted imaging, *T2W* T2-weighted imaging

Cranial magnetic resonance imaging of these patients revealed meningeal infiltration and cortical laminar necrosis in the right parietal lobe, effacement and slight expansion in the sulci in case 1 (Fig. 2A); dural contrast enhancement in case 2; acute diffusion restriction consistent with ischemia at the cervicomedullary junction and increased T2A/FLAIR signal in this area in case 3 (Fig. 2B); cerebral ischemic lesions in white matter and left internal capsule and hemorrhagic transformation in case 4 (Fig. 2C); diffuse thrombosis in the venous sinuses in case 5 (Fig. 2D); severe stenosis of the cerebral sinuses secondary to intracranial hypertension and increased cerebrospinal fluid distance in the optic nerve sheath in case 6 (Fig. 2E); and dural thickening and hyperintense lesions in the spinal cord on spinal MRI during the initial neurological involvement in case 7 who had a relapse 6 months later, with signal changes in the cortical gray matter and white matter in the left frontotemporal region, along with pial and dural contrast enhancement at this level, granulomatous meningeal involvement-like appearance, and chronic lacunar infarcts in the inferior part of the cerebellar hemisphere. In his second relapse, which occurred 74 months later, there were increased signals in the subcortical white matter in both parietal lobes, end artery ischemia, sequelae in the vascular territory of the PICA in the left cerebellar hemisphere, and an infarct in the left frontoparietal internal carotid artery territory area. In the third relapse, 19 months later, there was dural contrast enhancement and a widespread syringomyelia cavity in the cervical and dorsal spinal cord on MRI (Fig. 2F)).

Cerebrospinal fluid (CSF) analysis was available in 2 of these patients. In Case 6, the CSF revealed elevated intracranial pressure (66 cm H2O), low glucose levels (56 mg/dL), and the presence of type 2 oligoclonal bands. In Case 5, the only abnormal finding in the CSF was a mildly elevated protein level (48 mg/dL).

Among the 7 patients, 5 patients were treated with CYC and high dose GC for induction treatment. One patient who was already on CYC treatment at the occurrence of CNS involvement, received only IVIG treatment due to suspicion of meningitis, and 1 patient with cerebral venous sinus thrombosis was treated with RTX and high dose GC for induction. Maintenance treatment of the 6 patients who did not experience a relapse included both RTX and MMF in 2 patients, RTX in 2, both RTX and AZA in 1 patient, and mepolizumab in 1 patient. The last patient (case 7) was treated with alternating oral and intravenous CYC for 23 months due to refractory AAV manifestations other than CNS involvement. The first neurologic involvement presented as hypertrophic pachymeningitis 23 months after diagnosis, and high-dose GC and CYC treatment was administered. After 6 cycles of monthly intravenous CYC, he experienced a relapse with meningeal involvement and a lacunar infarct in the cerebellar hemisphere. He received high dose GC and CYC dose was increased by 50%. Following 74 months of alternating oral and intravenous CYC treatment, he had a relapse with a frontoparietal infarct. CYC was stopped, and RTX, AZA, and high dose GC treatment were initiated. 19 months later, he had another relapse with meningeal involvement, and secondary syringomyelia was detected. IVIG and high dose GC were started. He was relapse-free with RTX, IVIG, AZA, and low dose GC for 3 years. Then, he was lost to follow-up and died of an unknown cause, 16 years after CNS involvement.

Overall, 6 patients were still alive for a mean (SD) duration of 38 (24.1) months after AAV diagnosis and for a mean (SD) duration of 27.8 (26.9) months after CNS involvement. The last patient (Case 7) who had died was the only patient who experienced relapses of CNS involvement. The other patients experienced only one episode of CNS involvement, but except for one patient, all of them recovered with sequela including epilepsy, blurred vision, muscle weakness, numbness, or hearing loss, each affecting one patient. The demographics, AAV manifestations, neurologic symptoms, management, and outcome of these 7 patients are presented in Table 2.

**Table 2:** Demographic and clinical characteristics of AAV patients with CNS involvement

Case	Sex	Type of AAV	ANCA type	Age at AAV diagnosis	Age at CNS involvement	Other AAV manifestations	BVAS at CNS involvement	Neurologic signs or symptoms	Type of CNS involvement	Treatment just before CNS involvement	Treatment for CNS involvement	Outcome
1	W	GPA	P-ANCA	55	55	ENT, chest	13	Seizure, numbness	Meningeal involvement and ischemic CVA	N/A	CYC, high dose GC	Recovered with sequela
2	M	GPA	PR3-ANCA	41	42	Cutaneous, PNS	8	Headache, blurred vision, seizure	Meningeal involvement	CYC	RTX and IVIG	Recovered with sequela
3	M	GPA	PR3-ANCA	45	47	Cutaneous, mucous membrane, eye, chest, renal	11	Muscle weakness	Ischemic CVA	RTX, low dose GC	CYC, high dose GC	Recovered with sequela
4	W	EGPA	MPO-ANCA	42	42	Chest, CV, PNS	19	Muscle weakness, numbness	Ischemic CVA with hemorrhagic transformation	N/A	CYC, high dose GC	Recovered with sequela
5	M	MPA	MPO-ANCA	20	21	Eye, chest, renal	12	Headache, blurred vision	Cerebral venous sinus thrombosis	AZA, low dose GC	RTX, high dose GC	Recovered
6	W	GPA	C-ANCA	23	24	Eye, ENT, chest, renal	10	Headache, hearing loss	Intracranial hypertension	AZA	CYC, high dose GC	Recovered with sequela
7	M	GPA	C-ANCA	51	53	ENT, chest, renal, PNS	10	Muscle weakness, seizure, numbness	Meningeal, parenchymal involvement, secondary syringomyelia	CYC, low dose GC	CYC high dose GC	Exitus

*AAV* ANCA–associated vasculitis, *AZA* azathioprine, *BVAS* Birmingham Vasculitis Activity Score, *CNS* central nervous system, *CV* cardiovascular, *CVA* cerebrovascular accident, *CYC* cyclophosphamide, *EGPA* eosinophilic granulomatosis with polyangiitis, *ENT* ear nose throat, *F* female, *GC* glucocorticoids, *GI* gastrointenstinal tract, *GPA* granulomatosis with polyangiitis, *IVIG* intravenous immunoglobulin, *M* male, *MM* mucous membranes, *MPA* microscopic polyangiitis, *N/A* not applicable, *PNS* peripheral nerve system involvement, *RTX* Rituximab

*Constitutional symptoms: fever, arthralgias, myalgias, arthritis

### AAV manifestations mimicking CNS involvement

Among the 30 (49.1%) patients who presented with neurologic symptoms suggesting CNS involvement, neurologic work-up revealed that their symptoms were due to AAV manifestations other than CNS involvement. Table 3 shows the demographic features, type of AAV, ANCA status, symptoms or signs that led to a CNS work-up, AAV manifestations other than CNS involvement that contributed to these symptoms or signs, and the outcome. The most common AAV manifestation mimicking CNS involvement was PNS involvement and, the symptoms were numbness, muscle weakness, or headache. All of these 30 patients were treated with immunosuppressives and 23 of them recovered without sequela. 4 patients with PNS involvement recovered with some residual numbness. Blurred vision persisted in the patient with ocular involvement and in the 2 patients with orbital involvement.

**Table 3:** Patients with neurologic signs or symptoms caused by other AAV manifestations

Case	Sex, age	Type of AAV	Type of ANCA	Neurologic signs or symptoms	AAV manifestations mimicking CNS involvement	Outcome
8*	37, M	GPA	PR3-ANCA	Numbness	Peripheral nervous system involvement	Recovered with sequela
9	46, M	EGPA	MPO-ANCA	Numbness, muscle weakness	Peripheral nervous system involvement	Recovered with sequela
10	66, M	MPA	P-ANCA	Numbness	Peripheral nervous system involvement	Recovered with sequela
11	68, M	GPA	PR3-ANCA	Numbness	Peripheral nervous system involvement	Recovered with sequela
12	58, M	GPA	PR3-ANCA	Numbness	Peripheral nervous system involvement	Recovered with sequela
13	60, M	GPA	PR3-ANCA	Numbness	Peripheral nervous system involvement	Recovered**
14	30, F	GPA	P-ANCA	Headache, numbness	Peripheral nervous system involvement	Recovered with sequela
15	56, M	MPA	MPO-ANCA	Numbness, muscle weakness	Peripheral nervous system involvement	Recovered with sequela
16	32, M	GPA	PR3-ANCA	Headache, hearing loss	Frontal, maxillary and sphenoidal sinus involvement	Recovered with sequela
17	40, M	GPA	PR3-ANCA	Headache, hearing loss	Sphenoidal sinus involvement	Recovered
18	32, F	EGPA	MPO-ANCA	Headache, muscle weakness	Sinonasal involvement	Recovered
19	47, M	EGPA	MPO-ANCA	Headache, muscle weakness	Frontal sinus involvement	Recovered
20	25, F	GPA	C-ANCA	Headache	Nasal involvement	Recovered
21	67, F	GPA	C-ANCA	Dizziness and hearing loss	Nasal involvement	Recovered
22	53, M	GPA	C-ANCA	Headache, cough, fatigue	Disease activity (lung involvement)	Recovered with sequela
23	57, M	GPA	C-ANCA	Dizziness, Muscle weakness	Disease activity (lung involvement)	Recovered with sequela
24	23, M	GPA	C-ANCA	Diplopia, blurred vision, headache	Orbital and paranasal involvement, secondary facial paralysis and PNS	Recovered with sequela
25	61, F	GPA	C-ANCA	Blurred vision, hearing loss, dizziness	Orbital and paranasal involvement, secondary facial paralysis and PNS	Recovered with sequela
26	66, M	GPA	C-ANCA	Blurred vision	Orbital involvement	Recovered with sequela
27	53, F	GPA	C-ANCA	Headache, visual blurriness.	Ocular involvement (blepharitis and keratitis)	Recovered with sequela
28	31, M	GPA	C-ANCA	Paralysis of the facial muscles	Facial paralysis due to facial nerve involvement	Recovered with sequela
29	49, M	GPA	C-ANCA	Paralysis of the facial muscles	Facial paralysis due to orbital involvement	Recovered with sequela
30	42, F	GPA	C-ANCA	Paralysis of the facial muscles	Facial paralysis due to parotid gland involvement	Recovered with sequela
31	78, F	GPA	C-ANCA	Numbness, muscle weakness	Disease activity and PNS involvement	Recovered with sequela
32	45, F	GPA	C-ANCA	Numbness, headache	Disease activity and cervical hernia	Recovered
33	58, F	MPA	MPO-ANCA	Blurred vision	Retinal vasculitis	Recovered with sequela
34	24, F	GPA	C-ANCA	Blurred vision	Nasopharyngeal mass and PNS involvement	Recovered with sequela
35	68, F	GPA	C-ANCA	Headache	Parotid gland involvement	Recovered with sequela
36	65, M	EGPA	MPO-ANCA	Muscle weakness, headache	Cardioembolic CVA due to cardiac mass	Recovered with sequela
37	47, M	EGPA	MPO-ANCA	Muscle weakness, headache	Cardioembolic CVA due to cardiac thrombus	Recovered with sequela

*AAV* ANCA–associated vasculitis, *CVA* cerebrovascular accident, *EGPA* eosinophilic granulomatosis with polyangiitis, *GPA* granulomatosis with polyangiitis, *M* male, *MM* mucous membranes, *MPA* microscopic polyangiitis, *PNS* peripheral nerve system involvement

*These patients are numbered starting from 8 in order to avoid confusion with the 7 patients with actual CNS involvement of AAV, **Recovery was defined as the complete resolution of all neurologic symptoms and signs following immunosuppressive treatment in AAV patients with CNS involvement

### Drug-related adverse events and comorbidities mimicking CNS involvement

Neurologic symptoms were due to drug-related adverse events or comorbidities in 15 (24.5%) patients. Cardiovascular events were the leading secondary comorbidity (n = 8), followed by infections (n = 4), drug-related adverse events (n = 2), and posterior reversible encephalopathy syndrome (PRES) (n = 1). Among the 8 patients with cardiovascular disease, 3 recovered completely with anticoagulant and cardiac treatments, whereas 3 patients recovered with residual muscle weakness. One patient with hemorrhagic CVA secondary to hypertension and cardioembolic CVA secondary to atrial fibrillation recovered without sequelae. In the last patient with muscle weakness, symptoms regressed after treatment for heart failure.

Among the 4 patients whose neurologic symptoms were related to infections, one patient with spondylodiscitis complicated with aortic pseudoaneurysm, one with septic emboli and one with diabetic ketoacidosis complicated with pneumosepsis had died. The patient with skull base osteomyelitis recovered with a sequela of blindness. Among the 2 patients whose neurologic symptoms were drug-related adverse events, dose reduction of CYC in 1 patient and of glucocorticoid in 1 patient was sufficient to eliminate their symptoms. The patient diagnosed with PRES recovered with epilepsy sequelae.

### CNS findings with unclear etiology

Neurologic work-up did not lead to an underlying condition in the remaining 9 (14.7%) patients. Neurologic symptoms were transient in four patients, and did not relapse during follow-up periods of 36, 52, 57, and 120 months. In three patients, it was not possible to clearly differentiate CNS involvement from other pathologies.

The first patient, who presented with coma, died suddenly and cranial infection or CNS involvement could not be diagnosed. The second patient, who presented with syncope and later developed sequelae of muscle weakness, had a delayed presentation and concomitant acute kidney injury, making it difficult to make a clear distinction, possibly indicating a transient ischemic attack. The third patient, who presented with muscle weakness and imbalance, recovered with sequelae of muscle weakness after receiving immunosuppressive therapy. The remaining two patients were lost to follow-up after they visited our clinic with neurologic symptoms.

## Discussion

In this study, we focused on AAV patients who presented with neurologic symptoms and underwent evaluation for CNS involvement. Among the 430 AAV patients, 61 (14%) presented with neurologic symptoms suggesting CNS involvement. CNS involvement was quite rare, observed in only 7 (1.6%) patients. CNS involvement was the initial manifestation in 2 patients, while it developed within 3 years after diagnosis in the remaining 5 patients. Three relapses were observed in 1 patient. Five of them had a diagnosis of GPA and all patients had active disease at the time of CNS involvement. Mimickers of CNS involvement were relatively common. Among the 54 patients without CNS involvement, 30 (49.1%) patients had neurologic symptoms due to AAV manifestations other than CNS involvement and 15 (24.5%) due to secondary complications including drug-related adverse events and comorbidities. Cardiovascular diseases (n = 8) and infections (n = 4) were the most common causes of secondary complications. In the remaining 9 patients, we could not determine the exact underlying condition for their neurologic symptoms.

There were six previous studies on the frequency of CNS involvement in AAV patients that reported details of AAV patients with CNS involvement (Table 4). The frequency of CNS involvement among AAV patients varied across studies, ranging from 4.8 to 17.3% [12–15, 19]. Four studies examined 83, 77, 263, and 61 patients with GPA, reporting frequencies of CNS involvement as 4.8, 11.7, 3, and 16.4%, respectively [12–15]. The frequency of CNS involvement in MPA was reported as 7.7% in a cohort of 325 patients [14], and as 10.7% in another cohort that included 93 patients [12]. The frequency of CNS involvement among EGPA patients was 4.6% among 456 patients in one study [16], 16% among 25 patients in another study [12] and as 17.3% among 110 patients in a third study [19]. In our study, we found a lower frequency of 1.6% among our 430 AAV patients, which may be attributed to our strict approach for case ascertainment, for including only patients who are very likely to have CNS involvement. Despite the heterogeneity across the limited number of studies, CNS involvement seems to be a rare manifestation of AAV, irrespective of the type of AAV.

**Table 4:** Six previous studies on CNS involvement in AAV patients

	Tian Ma et al.	Zhang et al.	Fragoulis et al.	Huang et al.	Andre et al.^a^	Liu et al.	Current study
Total number of AAV patients	408	179	77	263	456^b^	110	61
Type of AAV, n							
GPA	83	61	77	263	N/A	N/A	41
MPA	325	93	N/A	N/A	N/A	N/A	11
EGPA		25	N/A	N/A	456	110	9
Frequency of CNS involvement, n (%)							
GPA	4 (4.8)	10 (16.4)	9 (11.7)	8 (3)	N/A	N/A	5 (12.1)
MPA	25 (7.7)	10 (10.7)	N/A	N/A	N/A	N/A	1 (9)
EGPA	N/A	4 (16)	N/A	N/A	21 (4.6%) A total of 88	19 (17.3)	1 (11)
CNS involvement definition	CNS manifestations present concomitant with vasculitis or occurring during AAV relapse, and responding to vasculitis treatment	NR	CNS symptomatology thought to be related to GPA if fulfilled the following conditions: coincided with AAV onset or new disease flare	NR	CNS manifestations present concomitant with vasculitis or occurring during AAV relapse, and responding to vasculitis treatment	CNS manifestations present concomitant with vasculitis and responding to vasculitis treatment	Neurological symptoms or signs concomitant with vasculitis, not explained by other pathologies
Exclusion criteria	Patients with secondary vasculitis, anti-GBM antibodies, SLE, RA, IBD, drug-induced vasculitis, or coexistence of other renal diseases	NR	Other conventional causes of CNS nosology, including but not confined to uncontrolled hypertension	NR	NR	Other CNS disease-related risk factors, including hypertension, hyperlipidemia, hyperglycemia, smoking, infection, and genetic factors	Peripheral cranial nerve involvement as a result of upper respiratory tract or eye involvement
Cranial nerve involvement as a CNS involvement	NR	No	Yes	Yes	Yes	No	No
Characteristics of CNS involvement (n)	Vascular lesions (24 ischemic; 4 hemorrhagic) Pituitary mass (1)	Vascular (1 hemorrhagic) Arachnoid hemorrhage,coma and neurocognitive deficits (4)^d^ Meningeal (1) Encephalopathy (7) Pituitary (1) Cerebrovascular neuropathy (10)	Vascular (ischemic 5) Meningeal (2) Mastoiditis causing facial nerve palsy bilaterally (1) Orbital mass formation (1)	Vascular(3 hemorrhagic) Meningeal (4)^c^ Optic nerve compression secondary to orbital mass (2)	Vascular (46 Ischemic; 21 hemorrhagic) Meningeal (4) Neuropaties (18 cranial nerve palsies; 15 optic neuritis) Central retinal artery occlusion (9)	Vascular (12 ischemic; 3 hemorrhagic) Meningeal (2) PRES (7) Spinal cord involvement (3) Medulla oblongata involvement (3) Cerebellar ataxia (2)	Vascular (2) Meningeal (3^e^) Cerebral venous sinus thrombosis (1) Intracranial hypertension (1)
Frequency of CNS involvement at AAV diagnosis n/N, (%)	18/29 (62)	NR	3/9 (33)	5/8 (62.5)	71/83 (86)	NR	2/7 (28.5)
Mean time to development of CNS involvement	12 months (range 9–31)	NR	12 months (range 0–95)	NR	24 months (range 3–186)	35 months (range 1–132)	10 months (range 0–23)
BVAS at CNS involvement	Mean (SD) 23.5 ± 5.3	All had active disease, but mean BVAS was not reported	Median 12 (range 3–26)	All had ‘severe disease’, but mean BVAS/WG was not reported	NR	Median 16 (IQR: 14–22)	Median 11.8 (IQR 10–12.5)
Relapse rate of CNS involvement, n/N (%)	0/29	NR	1/9 (11.1)	NR	6/81 (7%)	7/19 (36.8)	1/7 (14.2)

*AAV* ANCA associated vasculitis, *BVAS* Birmingham Vasculitis Activity Score, *CNS* central nervous system, *BVAS/WG* Birmingham Vasculitis Activity Score/Wegener’s granulomatosis, *EGPA* eosinophilic granulomatosis with polyangiitis, *GBM* glomerular basement membrane, *GPA* granulomatosis with polyangiitis, *IBD* inflammatory bowel disease, *MPA* microscopic polyangiitis, *N/A* not applicable, *NR* not reported, *RA* rheumatoid arthritis, *SLE* systemic lupus erythematosus

^a^21 patients were included from French Vasculitis Study Group (FVSG) database, 5 patients from other centers, and 62 patients from the systemic literature. Frequency of CNS involvement at diagnosis and relapse rate of CNS involvement were reported among 83 and 81 patients, respectively

^b^French Vasculitis Study Group (FVSG) database

^c^More than one involvement was observed in one patient

^d^The neurological involvement of 4 patients diagnosed with EGPA was reported collectively

^e^2 of them had also vascular involvement

There were some methodological differences between these studies that made it challenging to compare the results and interpret the differences (Table 4). The definition of CNS involvement as well as exclusion criteria could not be obtained from some studies [12, 15]. While some authors included cranial nerve palsies as part of CNS involvement [13–16], others, including us, excluded cranial nerve palsies from the definition of CNS involvement [12, 19]. The reason for this was that cranial nerve palsies may be a manifestation of peripheral nerve involvement or caused by infiltration or compression by a granulomatous mass in the upper respiratory system or orbita. Although venous thrombosis is thought to be associated with systemic inflammation rather than vessel wall inflammation, we classified cerebral venous sinus thrombosis in a patient with active disease as CNS involvement [17]. Liu et al included 7 patients with PRES as CNS involvement, whereas the patient with PRES in our study was considered a complication due to severe hypertension. Two studies included patients whose neurological symptoms responded to immunosuppressive therapy in the definition of CNS involvement. However, this inclusion criterion may underestimate refractory patients. Finally, two studies examined CNS involvement among patients with GPA [13, 15], two studies, including ours, surveyed all types of AAV [12, 14], two studies reported CNS involvement among patients with EGPA [16, 19] and 1 study among patients with GPA and MPA. All of these differences contribute to the challenges in interpreting the frequency and characteristics of CNS involvement among AAV patients.

CNS involvement usually occurs early in the disease course. In the reported series, it was among the initial manifestations in 33–86% of AAV patients with CNS involvement [12–17]. In three studies including GPA patients with CNS involvement, it was present at disease onset in 51, 33, and 67% of the patients, respectively [13–15]. This rate was reported as 86% in a study with EGPA patients [17], and 62% in a study including both EGPA and MPA patients [14]. Overall, CNS involvement developed within 5 years after AAV diagnosis in patients who did not initially present with it, with a duration ranging from 12 to 60 months. In our study, CNS involvement was among the initial manifestations in 2/7 patients, while it developed within the first 3 years following diagnosis in the remaining 5 patients. All of the reported patients with CNS involvement, including ours, had active disease, with at least one more organ involvement at the same time. These results may suggest that CNS involvement is mostly observed in the early years of AAV and it is accompanied by another active organ involvement in all patients. Regarding relapse rate of CNS involvement, 2 studies did not provide any details on relapse [12, 15]. In one of the studies that included only GPA patients, the relapse rate was 1/9 (11%) [13]. There were no relapses among the 25 MPA patients [14], whereas 7% of 81 EGPA patients had a relapse. In the last study, which examined only EGPA patients, relapse was observed in 7 of 19 patients (36.8%) [19]. In our cohort, we observed only 1 patient with GPA who experienced three CNS relapses with different types of CNS involvement. However, since most of the studies defined relapse according to BVAS, it is not clear how many cases relapsed with CNS involvement.

Among the 61 patients with neurologic symptoms or signs suggesting CNS involvement, the most common cause was AAV involvement in organs or systems other than the CNS, such as ocular, orbital, and nasopharyngeal involvement (n = 30, 49.1%). The second common cause was complications such as infections and cardiovascular diseases (n = 15, 24.5%). Infections and cardiovascular diseases are among the most common causes of mortality in AAV patients, in addition to malignancy [18]. Cardiovascular diseases are the most important cause of death in most inflammatory rheumatic diseases. Accelerated atherosclerosis is frequently observed in patients with AAV and it is thought to develop either as a consequence of immunosuppression or associated with the underlying inflammatory disease [17]. Stroke risk and venous thromboembolism are also increased among AAV patients, especially during the early period of disease onset [20]. The incidence rate of stroke in AAV is higher than that in the general population. However, it is difficult to conclude whether these manifestations are part of vasculitic involvement or due to systemic inflammation [21]. In our two patients who experienced ischemic CVA, their young ages (42 and 47 years), absence of traditional cardiovascular risk factors, concomitant AAV activity in other systems at the time of the CVA, lack of pathological findings in the work-up for young stroke, and the presence of subacute and hemorrhagic acute ischemic lesions at different sites and time points on cranial imaging led us to attribute these events to small vessel vasculitis.

The main limitation of our study was its retrospective nature. Although, imaging findings were available for all of our patients, brain biopsy, which is the gold standard for the diagnosis of CNS involvement was lacking, and cerebrospinal fluid analysis was available in only two patients. Finally, one may find it challenging to include intracranial hypertension among the manifestations of CNS involvement in AAV. We considered that this was appropriate because AAV could contribute to increased intracranial pressure through subclinical inflammation, microvascular dysfunction, and venous outflow disturbances. We think that in an AAV patient, intracranial hypertension may be considered as a disease manifestation unless there is a more convincing explanation.

In conclusion, neurologic signs and symptoms suggesting CNS involvement were present in 14% of our 430 AAV patients. However, true CNS involvement was detected in only 11.5% of our AAV patients with neurologic symptoms. Overall, CNS involvement was uncommon, observed in only 1.6% of the entire cohort, leading to morbidity but not mortality. Non-CNS entities including ocular, orbital and nasopharyngeal involvement and complications such as PRES as well as complications like infections and cardiovascular disease, may mimic CNS involvement in patients with AAV and were more common than true CNS involvement. Thus, before intensifying immunosuppressive treatment in patients presenting with CNS symptoms, mimickers should be ruled out to minimize the potential harm associated with immunosuppression.

## Data Availability

All data relevant to the study are included in the article.

## Abbreviations

AAV: ANCA associated vasculitis
AZA: Azathioprine
CNS: central nervous system
CVA: cerebrovascular accident
CVST: cerebral venous sinus thrombosis
CYC: Cyclophosphamide
EGPA: eosinophilic granulomatosis with polyangiitis
ENT: ear: nose: throat
GC: glucocorticoids
GPA: granulomatosis with polyangiitis
IVIG: intravenous immunoglobulin
MPA: microscopic polyangiitis
PNS: peripheral nervous system
PRES: posterior reversible encephalopathy syndrome
RTX: Rituximab
